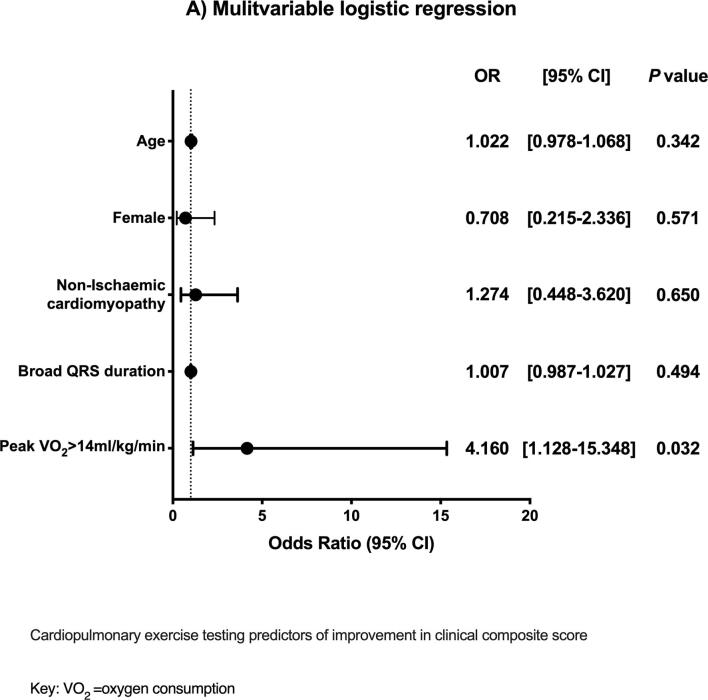# Clinical effectiveness of a dedicated cardiac resynchronization therapy pre-assessment clinic incorporating cardiac magnetic resonance imaging and cardiopulmonary exercise testing on patient selection and outcomes

**DOI:** 10.1016/j.ijcha.2021.100800

**Published:** 2021-06-09

**Authors:** Baldeep S. Sidhu, Justin Gould, Mark K. Elliott, Vishal S. Mehta, Steven A. Niederer, Gerald Carr-White, Christopher A. Rinaldi

**Affiliations:** a School of Biomedical Engineering and Imaging Sciences, King’s College London, UK; b Guy’s and St Thomas’ Hospital, London, UK

**Keywords:** Cardiopulmonary exercise testing, Cardiac magnetic resonance imaging, Cardiac resynchronization therapy, Heart failure

## Abstract

**Background:**

Pre-procedural assessment of patients undergoing cardiac resynchronization therapy (CRT) is heterogenous and patients implanted with unfavorable characteristics may account for non-response. A dedicated CRT pre-assessment clinic (CRT PAC) was developed to standardize the review process and undertake structured pre-procedural evaluation. The aim of this analysis was to determine the effectiveness on patient selection and outcomes.

**Methods:**

A prospective database of consecutive patients attending the CRT PAC between 2013 and 2018 was analyzed. Pre-operative assessment included cardiac magnetic resonance (CMR) and cardiopulmonary exercise testing (CPET). Patients were considered CRT responders based on improvement in clinical composite score (CCS) and/or reduction in left ventricular end-systolic volume (LVESV) ≥ 15% at 6-months follow-up.

**Results:**

Of 252 patients reviewed in the CRT PAC during the analysis period, 192 fulfilled consensus guidelines for implantation. Of the patients receiving CRT, 82% showed improvement in their CCS and 57% had a reduction in LVESV ≥ 15%. The presence of subendocardial scar on CMR and a peak VO_2_ ≤ 12 ml/kg/min on CPET predicted CRT non-response. Two patients were unsuitable for CRT as they had end-stage heart failure and died during follow-up. The majority of patients initially deemed unsuitable for CRT did not suffer from unexpected hospitalization for decompensated heart failure or died from cardiovascular disease; only 8 patients (13%) received CRT devices during follow-up because of symptomatic left ventricular systolic impairment.

**Conclusion:**

A dedicated CRT PAC is able to appropriately select patients for CRT. Pre-procedural investigation/imaging can identify patients unlikely to respond to, or may not yet be suitable for CRT.

## 1 Introduction

Cardiac resynchronization therapy (CRT) improves heart failure morbidity and mortality however 30–40% of patients fail to benefit [1], [2], [3], [4]. Non-response may be multifactorial related to both patient selection and CRT implantation and delivery. Mullens *et al.* have previously described a post-implantation CRT optimization clinic to investigate the causes of CRT non-response [5]. In 75 consecutive patients with persistent symptomatic heart failure multiple factors were identified including anemia, suboptimal medical therapy, underlying narrow QRS duration and primary right ventricular dysfunction. Importantly many of these factors may be identified pre-implantation and prospective identification of predictors of CRT non-response may both improve outcomes and avoid implantation in ineligible patients [6]. We have introduced a bespoke CRT pre-assessment clinic (CRT PAC) to standardize the review process for patients considered for CRT and identify patients with unfavorable characteristics (including cardiac magnetic resonance (CMR) to assess myocardial scar) and ensure patients satisfied consensus guidelines for CRT implantation [1], [2]. We have previously demonstrated the economic benefits of this bespoke approach [7]. The aim of this analysis was to determine the clinical benefit of the CRT PAC and the benefit of pre-procedural investigation/imaging. We assessed the outcomes in patients deemed eligible for CRT going through the clinic in terms of clinical and echocardiographic response to CRT.

## 2 Methods

All patients had previously been assessed in an outpatient consultant led cardiology clinic where CRT was felt appropriate and a referral made for implantation. A prospective database of consecutive patients attending the CRT PAC at Guy’s and St Thomas’ NHS Foundation Trust, UK between 2013 and 2018 was analyzed. Patients underwent the following investigations (where appropriate); blood tests, electrocardiogram, echocardiogram, CMR with late gadolinium enhancement imaging, cardiopulmonary exercise test (CPET), 6-minute walk test and Minnesota Living with Heart Failure Questionnaire (MLWHFQ). The left ventricular ejection fraction (LVEF) used for CRT decisions was based on two-dimensional echocardiography (biplane Simpson’s rule) rather than CMR [1], [2]. Following investigations, all patients were reviewed by a cardiologist with a specialist interest in heart failure where a final decision regarding device therapy was made. Patients who were New York Heart Association functional class IV were offered a pacemaker rather than a defibrillator due to their poor prognosis and were also given a pacemaker if they declined a defibrillator. Patients felt to be unsuitable for CRT were followed-up in the CRT PAC as previously described [7]. CRT response was assessed after six-months of follow-up using (A) clinical composite score (CCS) consisting of alive, no hospitalizations with decompensated heart failure, improvement in ≥ 1 New York Heart Association (NYHA) functional class or improvement in global assessment [8], [9] and (B) change in left ventricular end-systolic volume (LVESV) ≥ 15%. The study received institutional approval from Guys and St Thomas’ Hospital.

### 2.1 Statistical analysis

Results are presented as mean ± standard deviation for normally distributed variables and as median (interquartile range (IQR)) for non-normally distributed variables. When investigating the change from baseline variables a paired sample *t*-test was used for normally distributed data and for non-normally distributed data a Wilcoxon signed-rank test. Univariable and multivariable binary logistic regression was performed to determine predictors of CRT response. Variables statistically significant at univariable analysis as well as important clinical covariables were used as the basis for multivariable analysis. A *P-*value < 0.05 was statistically significant. Statistical analyses were performed using Prism (GraphPad Software Inc., Version 7, CA) and SPSS (IBM Switzerland, Version 25, Switzerland).

## 3 Results

### 3.1 Study population

Between September 2013 and June 2018 a total of 252 patients were seen in the CRT PAC. Baseline demographics are provided in Table 1. Patients were 70.6 ± 10.8 years old, predominantly male (72.6%) with an even distribution of ischemic (50.4%) and non-ischemic cardiomyopathy (49.6%). The mean NYHA functional class was 2.5 ± 0.6, QRS duration was 157.1 ± 28.2 ms and LVEF 31.9 ± 10.1%. Patients with ischemic versus non-ischemic cardiomyopathy were more likely to be male, have diabetes and have a more severely dilated and impaired left ventricle.

**Table 1:** Baseline patient demographics.

Variable	Total (N = 252)	Ischaemic cardiomyopathy (N = 127)	Non-ischaemic cardiomyopathy (N = 125)	*P-*value
Age, ±SD	70.6 ± 10.8	71.8 ± 8.9	69.3 ± 12.3	0.232
Male, N(%)	183 (72.6)	107 (84.3)	76 (60.8)	<0.001
**Co-morbidities, N(%)**				
Coronary artery bypass grating	48 (19.0)	48 (37.8)	0 (0)	<0.001
Percutaneous coronary intervention	53 (21.0)	50 (39.4)	3 (2.4)	<0.001
Valve repair	25 (9.9)	8 (6.3)	17 (13.6)	0.053
Hypertension	89 (35.3)	49 (38.6)	40 (32.0)	0.276
Atrial Fibrillation	122 (48.4)	58 (45.7)	64 (51.2)	0.382
Diabetes Mellitus	72 (28.6)	44 (34.7)	28 (22.4)	0.031
Chronic obstructive pulmonary disease	24 (9.5)	11 (8.7)	13 (10.4)	0.640
Chronic kidney disease	60 (23.8)	33 (26.0)	27 (21.6)	0.416
>1 additional comorbidity not already listed	116 (46.0)	53 (41.7)	63 (50.4)	0.169
**Medications, N(%)**				
Angiotensin-converting enzyme inhibitor/Angiotensin receptor blocker	220 (87.3)	111 (87.4)	109 (87.2)	0.962
Beta-blockers	210 (83.3)	107 (84.3)	103 (82.4)	0.695
Mineralocorticoid receptor antagonist	113 (44.8)	65 (51.2)	48 (38.4)	0.042
Diuretic	147 (58.3)	80 (63.0)	67 (53.6)	0.132
Anti-arrhythmic	34 (13.5)	20 (15.7)	14 (11.2)	0.293
Anticoagulation	116 (46.0)	58 (45.7)	58 (46.4)	0.908
Statin	161 (83.9)	104 (81.9)	57 (45.6)	<0.001
New York Heart Association functional class, ±SD	2.5 ± 0.6	2.5 ± 0.6	2.4 ± 0.7	0.865
QRS duration, ±SD	157.1 ± 28.2	154.8 ± 28.8	159.3 ± 27.6	0.211
**QRS morphology, N(%)**				
Left bundle branch block	139 (55.1)	74 (58.3)	65 (52.0)	0.319
Right ventricular paced	69 (27.4)	25 (19.7)	44 (35.2)	0.006
Other	44 (17.5)	30 (22.1)	18 (12.8)	0.053
**2D Echocardiogram, N(%)**				
Left ventricular ejection fraction	31.9 ± 10.1	30.7 ± 10.0	33.1 ± 10.1	0.040
Left ventricular end-diastolic volume	189.8 ± 78.8	205.3 ± 69.3	174.8 ± 84.7	<0.001
Left ventricular end-systolic volume	130.5 ± 56.3	145.5 ± 59.2	115.7 ± 49.4	<0.001
Minnesota Living with Heart Failure Questionnaire, ±SD	47.2 ± 25.6	46.1 ± 24.5	48.3 ± 26.8	0.420
6 min walk test, ±SD	287.6 ± 136.2	287.0 ± 133.6	288.2 ± 139.6	0.854
**Blood results, ±SD**				
Haemoglobin	130 ± 18	128 ± 19	132 ± 15	0.065
Creatinine	123 ± 54	129 ± 52	117 ± 55	0.013
NT-proBNP	2866 ± 6525	3007 ± 5514	2712 ± 4629	0.445

### 3.2 Outcomes of patients attending CRT PAC

**Fig. 1:**
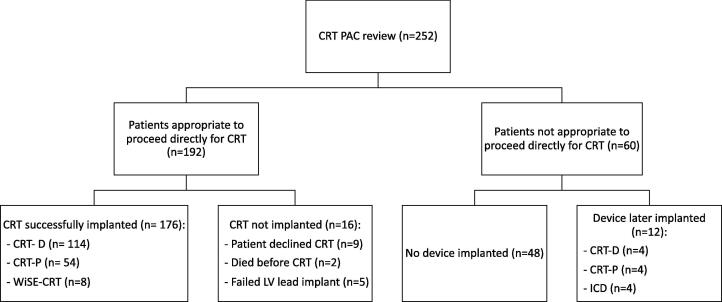
Flow-chart of patients attending the cardiac resynchronization therapy pre-assessment clinic.

192 (76.2%) patients were deemed eligible to undergo CRT (Fig. 1). Of the CRT eligible patients, 9 declined CRT and 2 died prior to the procedure. On an intention to treat basis of 192 patients, 5 (2.6%) had a failed left ventricular (LV) lead implant and 75 (39%) were upgrades. 78 received de novo CRT defibrillators (CRT-D), 15 de novo CRT pacemakers (CRT-P), and 8 WiSE-CRT (wireless LV endocardial pacing). The major complication rate was low at 1.1% due to the development of pericardial tamponade requiring pericardiocentesis, minor complications was 0.6% due to a pneumothorax requiring drainage and 1.1% of patients required a lead revision within the follow-up period.

### 3.3 Cardiac resynchronization therapy response rate

CRT response was assessed at a median of 6 months (IQR 6–8 months) (Table 2, Table 3). During this period, 3 (1.7%) patients were admitted to hospital with decompensated heart failure, 6 (3.4%) patients died and 2 (1.1%) patients were lost to follow-up. The mean increase in LVEF post CRT was 8.1 ± 10.7% (*P* < 0.001). There were statistically significant improvements in LVEF, LV end-diastolic volume, LVESV, NYHA functional class, 6-minute walk test, MLWHFQ and NT-proBNP (all *P* < 0.01) with CRT. Overall 82% improved their CCS and 57% had a reduction in LVESV ≥ 15%. In patients who underwent WiSE-CRT implantation, 1 died before review, 6/7 (85.7%) improved their NYHA functional class, 75% improved their CCS and 42.9% showed a reduction in LVESV ≥ 15%.

**Table 2:** Patient outcomes following CRT.

Variables	Before CRT	After CRT	*P-*value
New York Heart Association functional class, ± SD	2.5 ± 0.7	1.7 ± 0.7	<0.001
**2D Echocardiogram, N(%)**			
Left ventricular ejection fraction	29.3 ± 8.3	37.3 ± 12.4	<0.001
Left ventricular end-diastolic volume	205.1 ± 82.0	175.6 ± 57.7	<0.001
Left ventricular end-systolic volume	143.9 ± 53.5	118.0 ± 52.9	<0.001
Minnesota Living with Heart Failure Life Questionnaire, ± SD	44.0 ± 24.8	30.8 ± 25.8	<0.001^A^
6 min walk test, ± SD	312.3 ± 117.8	337.1 ± 120.3	0.006^A^
**Blood results, ± SD**			
Haemoglobin	132 ± 17	131 ± 18	0.196^B^
Creatinine	117 ± 42	119 ± 43	0.199^B^
NT-proBNP	2336 ± 2894	1929 ± 2767	0.002^C^

A- data for 86 patients.

B- data for 141 patients.

C- data for 108 patients.

**Table 3:** Patient outcomes according to whether patients had left bundle branch block or non-left bundle branch block.

Variables	Before CRT	After CRT	*P-*value
**Left bundle branch block**			
New York Heart Association functional class, ±SD	2.6 ± 0.7	1.6 ± 0.7	<0.001
**2D Echocardiogram, N(%)**			
Left ventricular ejection fraction	28.0 ± 6.9	36.6 ± 10.9	<0.001
Left ventricular end-diastolic volume	206.5 ± 64.3	175.3 ± 60.9	<0.001
Left ventricular end-systolic volume	149.1 ± 52.2	117.9 ± 54.1	<0.001
Minnesota Living with Heart Failure Life Questionnaire, ± SD	44.4 ± 26.2	28.8 ± 22.6	<0.001
6 min walk test, ± SD	322.0 ± 118.7	341.2 ± 112.2	0.108

**Non-left bundle branch block**			
New York Heart Association functional class, ±SD	2.6 ± 0.7	1.7 ± 0.7	<0.001
**2D Echocardiogram, N(%)**			
Left ventricular ejection fraction	30.6 ± 9.5	39.4 ± 13.8	<0.001
Left ventricular end-diastolic volume	202.5 ± 107.4	176.1 ± 52.2	0.004
Left ventricular end-systolic volume	134.8 ± 55.1	118.1 ± 51.2	0.002
Minnesota Living with Heart Failure Life Questionnaire, ± SD	43.5 ± 23.2	33.7 ± 29.6	0.024
6 min walk test, ± SD	297.8 ± 116.5	330.9 ± 113.4	0.018

### 3.4 Cardiac magnetic resonance imaging and predictors of CRT response

CMR was performed in 80/93 (86.0%) patients undergoing de novo CRT (excluding upgrades) (13 patients refused, were too large for the scanner or artefacts from metal implants rendered images non-diagnostic). Of patients undergoing CMR, 50% had an ischemic aetiology and were 70.4 ± 9.3 years old, predominantly male (75.0%) with a mean QRS duration 150.1 ± 19.9 ms and LVEF 29.0 ± 7.9%. Myocardial scar was identified in 49 (61.3%); sub-endocardial in 40, sub-epicardial in 1 and mid-wall fibrosis in 8. The presence of subendocardial scar was associated with a failure to improve CCS at univariable logistic regression (Odds ratio (OR) 5.063, 95% Confidence Interval (CI) 1.018–25.187; *P* = 0.048) and multivariable logistic regression (OR 6.715, 95% CI 1.153–39.090; *P* = 0.034) but was not associated with failure to reduce LVESV ≥ 15% (OR 2.267, 95% CI 0.841–6.111; *P* = 0.106). 22 patients had posterolateral scar (defined as ≥50% subendocardial scar in ≥1 of the following segments; basal posterior, basal posterolateral, mid posterior and mid posterolateral); 17 patients had the LV lead placed within scar (other locations were not anatomically viable) and 5 patients were paced outside scar (whereby the LV lead was placed in an anterior or anterolateral position). Pacing outside of scar vs. pacing within scar did not result in a significant improvement in CCS (80 vs. 77%; *P* = 1.000) or reduction in LVESV ≥ 15% (83 vs. 80%; *P* = 1.000).

### 3.5 Cardiopulmonary exercise testing and predictors of CRT response

**Fig. 2:**
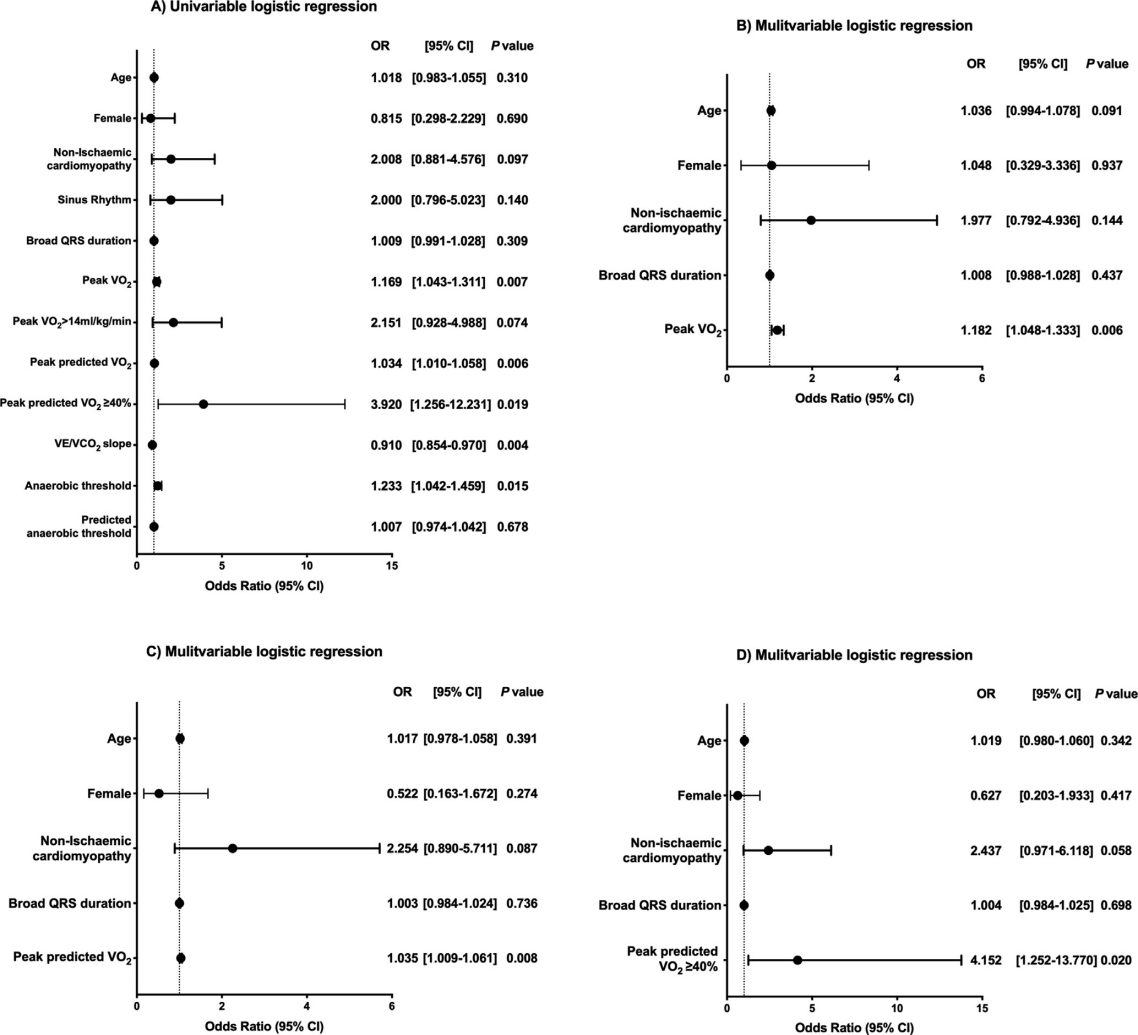
Cardiopulmonary exercise testing predictors of reduction in left ventricular systolic volume ≥ 15% Key: VCO_2_ = rate of elimination of carbon dioxide; VE = minute ventilation; VO_2_ = oxygen consumption.

**Fig. 3:**
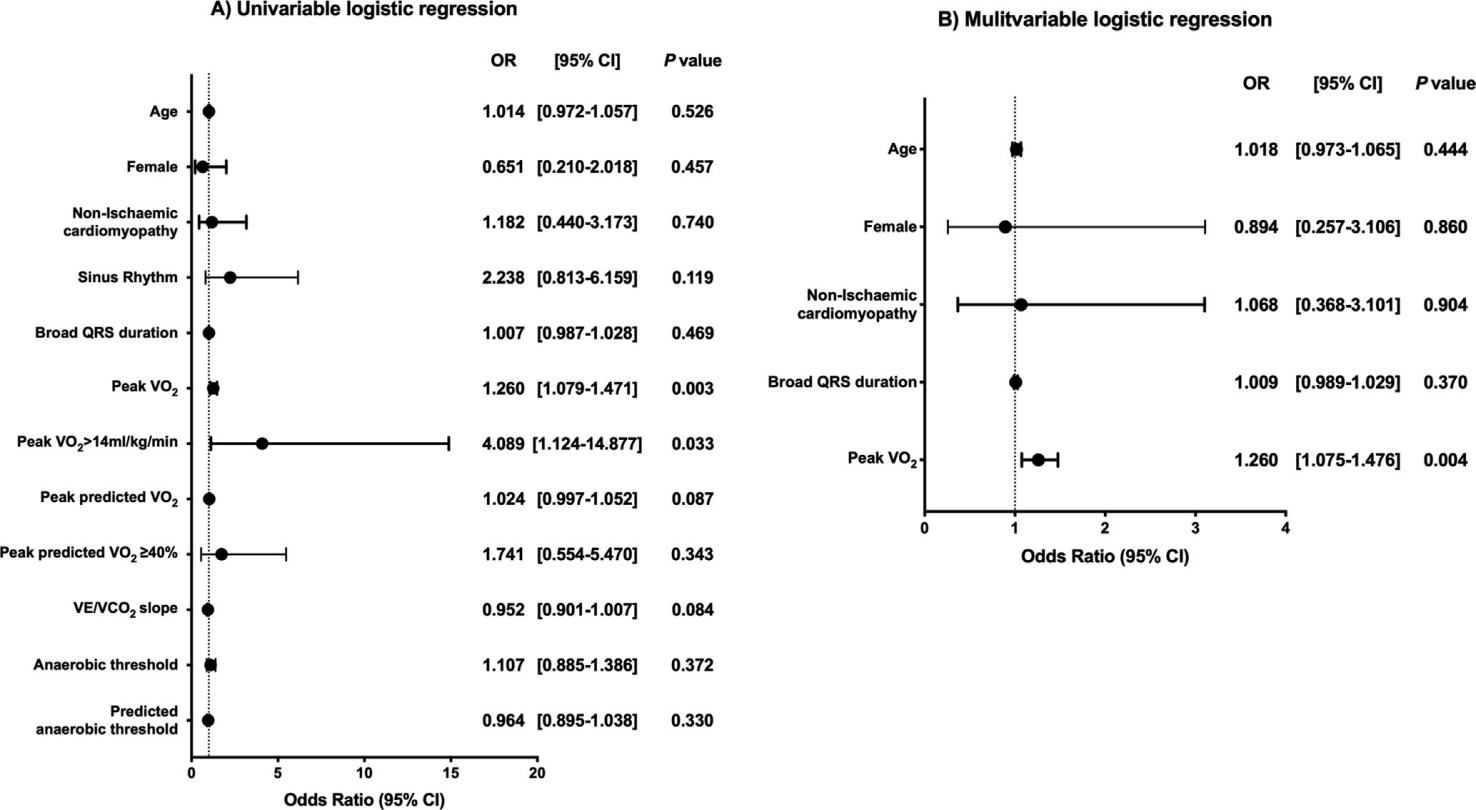
cardiopulmonary exercise testing predictors of improvement in clinical composite score key VCO2 = rate of elimination of carbon dioxide; VE = minute ventilation VO2 = oxygen comsumption.

Pre-procedural CPET was available in 126/176 (71.6%) patients (50 patients refused or were unable to carry out the exercise test) with a mean age of 68.6 ± 11.4 years old, 80.2% male, 44.4% non-ischaemic cardiomyopathy, 50.8% NYHA III-IV, 44.4% atrial fibrillation, mean QRS duration 163.2 ± 26.1 ms and LVEF 29.2 ± 8.0%. Predictors of improvement in CCS and LVESV ≥ 15% are provided in Fig. 2, Fig. 3.

**Supplementary figure 1:**
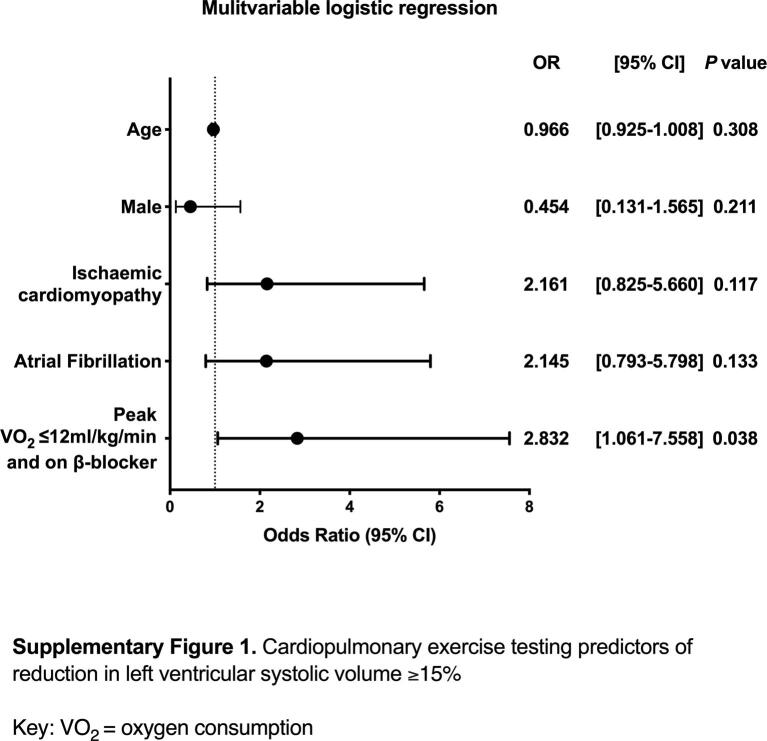


We investigated the outcomes of patients taking β-blockers (βB) who had a peak VO_2_ ≤ 12 ml/kg/min. A significantly higher proportion of patients with a peak VO_2_ ≤ 12 ml/kg/min vs. > 12 ml/kg/min had atrial fibrillation (59.1% vs. 34.8%; *P* = 0.018), NYHA III-IV (75% vs. 36.4%; *P* < 0.001), worse LVEF (28.0% vs 30.8%; *P* = 0.029) and were less likely to reach a respiratory exchange ratio (RER) > 1 (52.3% vs. 72.7%; *P* = 0.041). They were matched in terms of age (69.3 vs. 68.6 years; *P* = 0.976), non-ischaemic cardiomyopathy (43.2% vs. 48.5%; *P* = 0.697) and QRS duration (164.7 vs. 158.5 ms; *P* = 0.089). At both univariable and multivariable logistic regression, a peak VO_2_ ≤ 12 ml/kg/min in patients taking βB was associated with CRT non-response defined as an absence of improvement in CCS (OR 3.063, 95% CI 1.082–8.669; *P* = 0.035) and absence of increase in LVESV ≥ 15% (OR 2.832, 95% CI 1.061–7.558; *P* = 0.038) (Supplementary Fig. 1).

### 3.6 Outcome of patients initially felt unsuitable for CRT after pre-assessment review

**Fig. 4:**
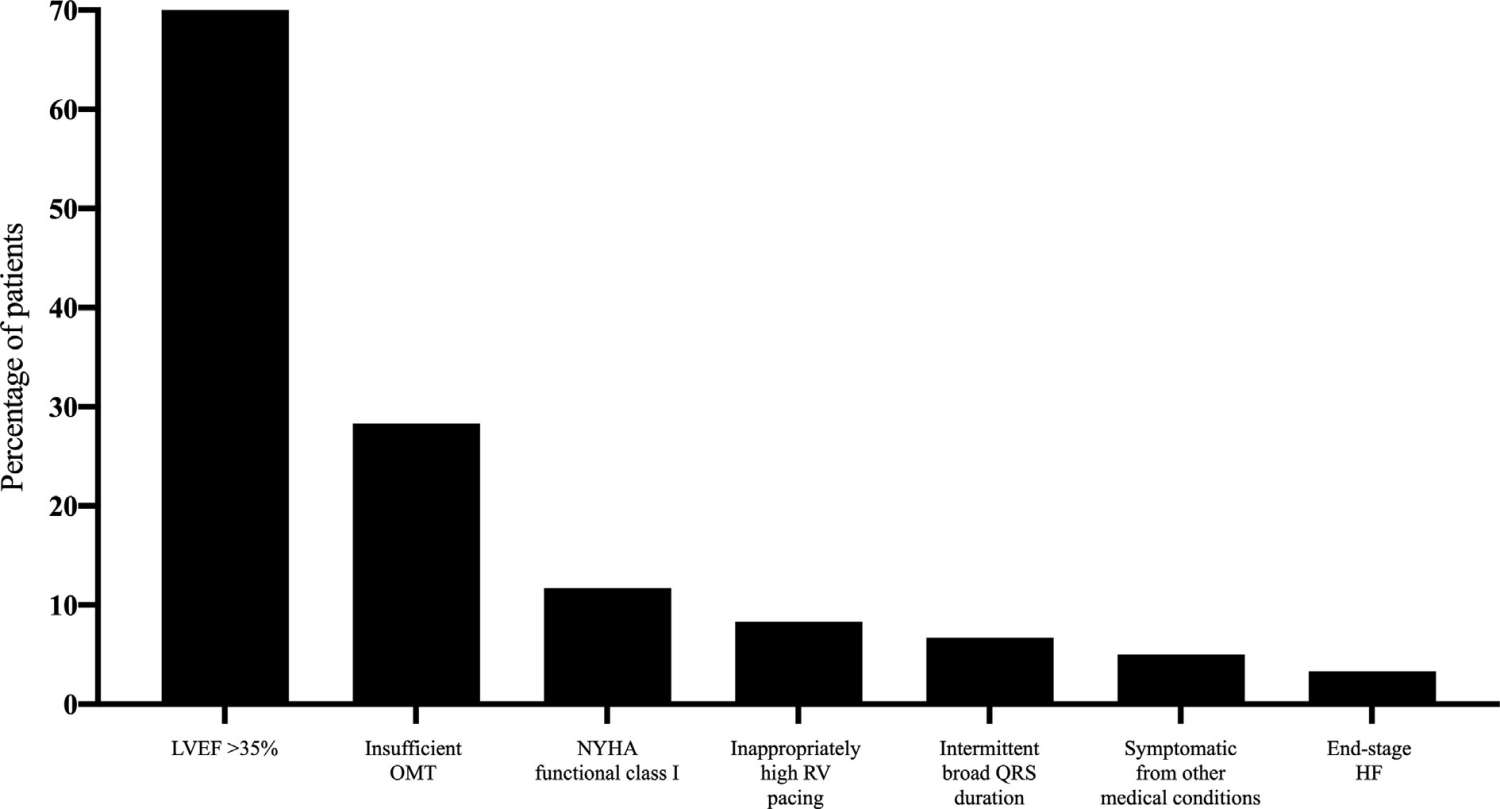
Reasons why patients were ineligible for CRT key: HF = heart failure; RBBB = right bundle branch block. RV = right ventricular.

As previously described [7], 60 (24%) patients were deemed ineligible to receive CRT often for a combination of reasons (Fig. 4). Eight patients underwent device implantation during follow-up as they became symptomatic or had persistent left ventricular systolic impairment despite medical optimization [7].

## 4 Discussion

We present outcomes from a dedicated and specialist CRT PAC. Studies have demonstrated that medical and device optimization can result in improved patient outcomes [5], [10]. However, translating these results into real-world clinical practice is difficult and outcomes are often far below those reported in clinical trials. We hypothesized a CRT PAC we would be able to appropriately apply evidence-based guidelines in a standardized manner and improve patient outcomes.

The main findings from the CRT PAC show:

1. 82% of patients who underwent CRT had improvement in their CCS and 57% had reduction in LVESV ≥ 15% after a median follow-up of 6 months.
2. CMR-identified myocardial scar and CPET predicted CRT non-response.

The CRT PAC ensured patients underwent relevant pre-procedural investigations immediately prior to intervention and ensured consensus guidelines were always followed. This allowed a thorough review of patients and ensured only those who were fully medically optimized and suitable for implantation proceeded to intervention.

### 4.1 A cardiac resynchronization therapy pre-assessment clinic appropriately selects patients

CRT non-response is defined heterogeneously in the literature, with some studies relying on evidence of reverse LV remodeling whilst others using a CCS [10]. Studies have shown differing patient outcomes when the CCS definition is applied [9], [11], [12]. A recent meta-analysis of three double-blind, randomized trials involving 1591 patients showed an overall 60% improvement in CCS at 6 months [13]. The improvement in CCS at 6 months in the current study of 82% compares favorably and additionally 57% showing an improvement in LVESV ≥ 15%. A potential benefit of a dedicated CRT PAC is the ability to identify patients that do not fulfil CRT implant criteria or who require further optimization prior to CRT [7]. In our analysis one quarter (24%) referred to the CRT PAC did not fulfil consensus guideline criteria for CRT and 8 (13.3%) patients subsequently underwent CRT during the follow-up period. Furthermore, 2 patients were identified as having end-stage heart failure and died. However, none of the remaining patients were admitted to hospital with decompensated heart failure, nor died from cardiovascular causes demonstrating that patients were appropriately identified and did not suffer unexpected adverse outcomes. This is important, as CRT may be harmful in patients who do not meet guideline defined criteria as shown in the ECHO-CRT study [6]. The commonest reason for finding a patient was unsuitable for CRT was an improvement in LVEF at CRT PAC review compared with their initial echocardiogram performed prior to referral to the CRT PAC (45.1 ± 7.1% vs. 34.1 ± 10.5%; *P* < 0.001). Guidelines recommend patients with chronic heart failure should be on optimal medical therapy for at least 3 months before considering CRT [1], [2]. We did not have a matched control group to compare but we can speculate that the favorable CRT response seen may be due to patient selection with non-implantation of patients ineligible to receive CRT.

### 4.2 Predictors of CRT response

#### 4.2.1 Cardiac magnetic resonance imaging

CMR is the preferred imaging modality to assess myocardial fibrosis and the aetiology underlying heart failure. The presence of myocardial scar is inversely proportional to reverse LV remodeling [14] and in keeping with this we found subendocardial scar was associated with CRT non-response. Studies have shown that placing the LV lead within posterolateral scar is associated with CRT non-response [15], [16]. Pre-procedural knowledge of scar in our cohort did not result in improved CRT response however implant strategies were not routinely performed using guidance strategies to avoid myocardial scar that was identified. Our results confirm the predictive value of CMR scar in CRT non-response and support the need for randomized studies to investigate whether image guidance avoiding myocardial scar can reliably improve CRT outcomes. Indeed, the ongoing multi-center randomized controlled trial investigating the benefit of CMR guided CRT implantation in ischaemic cardiomyopathy will provide important insights (NCT03992560).

#### 4.2.2 Cardiopulmonary exercise testing

CPET is a useful clinical adjunct to assess a patient’s cardiac reserve and functional capacity. In keeping with prior studies, clinical and echocardiographic responders were more likely to show better cardiopulmonary exercise capacity at baseline [17]. Guidelines recommend that in patients taking βB, a peak VO_2_ ≤ 12 ml/kg/min can be used as a cut-off to list patients for heart transplantation [2], [18]. In our cohort a peak VO_2_ ≤ 12 ml/kg/min was independently associated with an absence of clinical response and LV remodeling. At baseline these patients were more likely to be symptomatic, suffer from atrial fibrillation and less likely to achieve a RER > 1 suggesting their limitation to exercise is multifactorial rather than from pure cardiac disease and this may be a useful clinical adjunct identifying patients unlikely to respond to CRT which could be discussed in pre-procedural planning. Indeed, these patients should be closely followed-up to determine their progress and ensure they are thoroughly optimized or offered further intervention if appropriate.

## 5 Limitations

This is a single-center, observational study and is susceptible to the same limitations as for all prospectively collected data. The lack of a randomized control group means that findings are hypothesis generating rather than definitive. Follow-up was assessed at six months and it is unclear whether a longer period would produce similar findings. Although pre-procedural imaging was performed this was not used to systemically guide implant strategies and we cannot exclude the fact that knowledge of scar location may improve CRT response. This would need a randomized study and we are currently undertaking a multicenter study of CMR guidance to assess this (NCT03992560). Likewise the results of CPET did not dictate implantation strategy and this may merit further investigation. Overall, the total number of patients inappropriately implanted with CRT is unknown and is likely to vary from center to center. CPET’s often require experienced operators to perform the test reliably and are time consuming which may limit their role in routine pre-assessment clinics.

## 6 Conclusion

A CRT PAC is able to appropriately select patients for CRT and lead to favorable outcomes in the majority of patients implanted. Pre-procedural assessment including CMR and CPET can prospectively identify patients who are less likely to respond to CRT. Further evaluation is required to assess whether pre-procedural assessment is able to guide strategies to improve CRT response

## 7 Disclosures

The study was supported by the Wellcome/EPSRC Centre for Medical Engineering [WT203148/Z/16/Z]. BSS is funded by NIHR and has received speaker fees from EBR systems, outside of the submitted work. JG has received project funding from Rosetrees Trust, outside the submitted work. JG, MKE and VM have received fellowship funding from Abbott, outside of the submitted work. CAR receives research funding and/or consultation fees from Abbott, Medtronic, Boston Scientific and MicroPort outside of the submitted work.

## Declaration of Competing Interest

The authors declare that they have no known competing financial interests or personal relationships that could have appeared to influence the work reported in this paper.

## Figures and Tables

**Supplementary figure 2:**
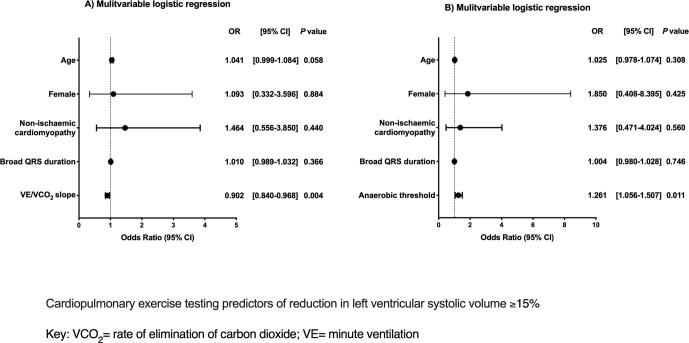


**Supplementary figure 3:**